# *Ixodes scapularis* Tick Saliva Proteins Sequentially Secreted Every 24 h during Blood Feeding

**DOI:** 10.1371/journal.pntd.0004323

**Published:** 2016-01-11

**Authors:** Tae Kwon Kim, Lucas Tirloni, Antônio F. M. Pinto, James Moresco, John R. Yates, Itabajara da Silva Vaz, Albert Mulenga

**Affiliations:** 1 Department of Veterinary Pathobiology, College of Veterinary Medicine, Texas A&M University, College Station, Texas, United States of America; 2 Centro de Biotecnologia, Universidade Federal do Rio Grande do Sul, Porto Alegre, Rio Grande do Sul, Brazil; 3 Centro de Pesquisas em Biologia Molecular e Funcional, Instituto Nacional de Ciência e Tecnologia em Tuberculose (INCT-TB), Pontifícia Universidade Católica do Rio Grande do Sul (PUCRS), Porto Alegre, Rio Grande do Sul, Brazil; 4 Department of Chemical Physiology, The Scripps Research Institute, La Jolla, California, United States of America; 5 Faculdade de Veterinária, Universidade Federal do Rio Grande do Sul, Porto Alegre, Rio Grande do Sul, Brazil; Johns Hopkins Bloomberg School of Public Health, UNITED STATES

## Abstract

*Ixodes scapularis* is the most medically important tick species and transmits five of the 14 reportable human tick borne disease (TBD) agents in the USA. This study describes LC-MS/MS identification of 582 tick- and 83 rabbit proteins in saliva of *I*. *scapularis* ticks that fed for 24, 48, 72, 96, and 120 h, as well as engorged but not detached (BD), and spontaneously detached (SD). The 582 tick proteins include proteases (5.7%), protease inhibitors (7.4%), unknown function proteins (22%), immunity/antimicrobial (2.6%), lipocalin (3.1%), heme/iron binding (2.6%), extracellular matrix/ cell adhesion (2.2%), oxidant metabolism/ detoxification (6%), transporter/ receptor related (3.2%), cytoskeletal (5.5%), and housekeeping-like (39.7%). Notable observations include: (i) tick saliva proteins of unknown function accounting for >33% of total protein content, (ii) 79% of proteases are metalloproteases, (iii) 13% (76/582) of proteins in this study were found in saliva of other tick species and, (iv) ticks apparently selectively inject functionally similar but unique proteins every 24 h, which we speculate is the tick's antigenic variation equivalent strategy to protect important tick feeding functions from host immune system. The host immune responses to proteins present in 24 h *I*. *scapularis* saliva will not be effective at later feeding stages. Rabbit proteins identified in our study suggest the tick's strategic use of host proteins to modulate the feeding site. Notably fibrinogen, which is central to blood clotting and wound healing, was detected in high abundance in BD and SD saliva, when the tick is preparing to terminate feeding and detach from the host. A remarkable tick adaptation is that the feeding lesion is completely healed when the tick detaches from the host. Does the tick concentrate fibrinogen at the feeding site to aide in promoting healing of the feeding lesion? Overall, these data provide broad insight into molecular mechanisms regulating different tick feeding phases. These data set the foundation for in depth *I*. *scapularis* tick feeding physiology and TBD transmission studies.

## Author Summary

*Ixodes scapularis*, the blacklegged tick or the deer tick, is among the most medically important ticks that transmit human tick borne disease agents including the spirochaete Lyme disease agent. To develop new tick control methods, a deeper understanding of how the tick feeds is needed. We collected saliva from rabbit fed blacklegged ticks every 24 h through five days of feeding and towards the end of feeding. We used novel proteomic technologies (Liquid Chromotography–Tandem Mass Spectrometry) to sequence and identify proteins in tick saliva. The main finding is that ticks apparently selectively inject functionally similar but unique proteins every 24 h, which we speculate is the tick's way to avoid the host's defense to protect important tick feeding functions from host immune system. The host immune responses to proteins present in 24 h *I*. *scapularis* saliva will not be effective at later feeding stages. This will influence how to design effective anti-tick vaccine antigens to stop disease agent transmission. This is the first comprehensive study of proteins in blacklegged tick saliva that provides insight into the molecular mechanisms that are at play at the tick feeding site every 24 h.

## Introduction

Ticks surpass all arthropods in transmission of a greater variety of pathogens including fungi, viruses, bacteria, and protozoa [1, 2]. In livestock production, ticks and tick-borne diseases (TBD) have caused annual losses in billions of US dollars globally [3, 4]. Recently, ticks have gained the attention in public health policy with a recent publication that advocated for One Health solutions listing 17 human TBDs among sources of human health concerns [5]. *Ixodes scapularis*, commonly known as the deer tick or blacklegged tick, is among the most medically important tick species and transmits 5 of the 14 human TBD agents in the USA: *Borrelia burgdorferi* [6], *Anaplasma phagocytophilum* [7], *Borrelia miyamotoi* [8], *Babesia microti* [9], and Powassan virus disease [10]. Likewise, close relatives of this tick including *I*. *pacificus* on the west coast of the USA and *I*. *ricinus* in Europe are vectors of important human TBD agents including *B*. *burgdorgferi*, *B*. *miyamotoi*, and *A*. *phagocytophilum* [11]. On this basis, the *I*. *scapularis* genome was sequenced [12, 13] and these data has provided opportunities for in depth studies of biological adaptations that make ticks successful vectors of pathogens. These data were postulated to facilitate studies that will reveal weaknesses that can be targeted for development of novel tick control methods [13].

In absence of effective vaccines against TBDs, controlling ticks using acaricides remains the most reliable method [14, 15]. Although effective in the short term, limitations of chemical acaricides such as selecting resistant tick populations, costs in new acaricide development, environmental and food contamination have necessitated the search for alternative tick control methods [4, 16, 17]. Immunization of animals has been advocated as a sustainable alternative tick control method [18]. The major limitation toward global adoption of anti-tick vaccines as an alternative tick control method is availability of effective target antigens. We are interested in understanding tick feeding physiology as a means to identify physiologically important proteins that can be targeted for anti-tick vaccine development.

Ticks accomplish feeding by lacerating the vasculature of the surrounding host tissue and sucking up blood that bleeds into the feeding lesion [19–21]. This destructive feeding style triggers the host defense mechanisms such as hemostasis, inflammation and immune responses. However, ticks counteract the host defense mechanisms by secreting pharmacologically active molecules in saliva to modulate host defenses [19, 20, 22–24]. In addition to blood meal acquisition, tick saliva proteins are also involved with the transmission and acquisition of TBD agents [25]. Reports of reduced pathogen transmission to repeatedly tick infested animals that developed resistance to tick feeding [26–29] provide credence to the importance of tick saliva proteins in vector tick competence. Thus, identification of tick saliva proteins will provide a basis for development of novel methods to interfere with tick feeding and prevention of pathogen transmission.

With the advent of next-generation sequencing (NGS) technologies, tick salivary gland transcriptomes have been described [23, 30–39]. However, the major limitation to these data is that it does not inform on which transcripts that encode for proteins are secreted in tick saliva. In an interesting approach to identify secreted tick salivary proteins (TSPs) Radulovic et. al., [40] and Lewis et. al., [41] used antibodies to 24–48 h tick saliva proteins [42, 43] to immunoscreen phage display cDNA expression libraries to identify 24–48 h *Amblyomma americanum* and 24 h *I*. *scapularis* immunogenic tick saliva proteins. Similar immunoscreening approaches were used to identify immunodominant *I*. *scapularis* tick saliva proteins [44–47]. In a related study, saliva of *I*. *scapularis* was analyzed by Edman degradation identifying 15 proteins [48]. Recently proteins in saliva of ixodid ticks from replete fed *Rhipicephalus sanguineus* [49], partial and replete fed *Rhipicephalus microplus* [50], three and five day fed *Dermacentor andersoni* [51], and replete fed adult and nymph *Haemaphysalis longicornis* [52] were identified. In argasid ticks, a lone study identified saliva proteins from twice fed *Ornithodoros moubata* ticks with saliva collected after 4 months from feeding [53]. Whereas studies reviewed here identified proteins in saliva of ticks at one or two feeding time points, this study has described proteins that *I*. *scapularis* ticks likely inject into animals every 24 h during the first five days of feeding and toward the end of the tick feeding process. The catalog of *I*. *scapularis* tick saliva proteins in this study provides an in depth view at protein families and/or molecular systems that are at play at the *I*. *scapularis* tick and host interface.

## Materials and Methods

### Ethics statement

All experiments were done according to the animal use protocol approved by Texas A&M University Institutional Animal Care and Use Committee (IACUC) (AUP 2011–207 and 2011–189) that meets all federal requirements, as defined in the Animal Welfare Act (AWA), the Public Health Service Policy (PHS), and the Humane Care and Use of Laboratory Animals.

### Ticks and saliva collection

*I*. *scapularis* ticks were purchased from the tick rearing facility at Oklahoma State University (Stillwater, OK, USA). Prior to feeding on rabbits, female ticks were paired with males to mate. Ticks were considered mated once males were detached from the females. Routinely, ticks were fed on rabbits as previously described [43]. Mated *I*. *scapularis* ticks were restricted to feed onto the outer part of the ear of New Zealand rabbits with orthopedic stockinet’s glued with Kamar adhesive (Kamar Products Inc., Zionsville, IN, USA). A total of 84 adult *I*. *scapularis* ticks (42 per ear) were placed into tick containment apparatus on three rabbits and allowed to attach.

To collect tick saliva, female ticks partially fed for 24 h (n = 43 ticks), 48 h (n = 40 ticks), 72 h (n = 40 ticks), 96 h (n = 40 ticks), 120 h (n = 40 ticks) as well as apparently fully fed but not detached from the host (BD, n = 8 ticks) and spontaneously detached ticks (SD, n = 6 ticks) were rinsed in Milli-Q water and dried on a paper towel. Rinsed ticks were placed dorsal-side down on double-sided tape on a glass slide. Salivation was induced by injecting 1–3 μL of 2% pilocarpine hydrochloride in phosphate buffered saline (PBS, pH 7.4) on the ventral side adjacent to the fourth leg coxa using a 34 gauge/ 0.5 inches/ 45° angle beveled needle on a model 701 Hamilton syringe (Hamilton Company, Reno, NV, USA). Subsequently, saliva was collected every 15–30 min using a Hamilton syringe for approximately 4h at room temperature.

### Protein digestion and sample preparation

Saliva of *I*. *scapularis* ticks (at least 2 μg total protein per run X3) for each specific feeding time point was digested in solution with trypsin. Saliva were diluted in 8 M urea/0.1 M Tris, pH 8.5, reduced with 5 mM Tris (2-carboxyethyl) phosphine hydrochloride (TCEP, Sigma-Aldrich, St Louis, MO, USA) and alkylated with 25 mM iodoaceamide (Sigma-Aldrich). Proteins were digested overnight at 37°C in 2 M urea/0.1M Tris pH 8.5, 1 mM CaCl_2_ with trypsin (Promega, Madison, WI, USA) with a final ratio of 1:20 (enzyme:substrate). Digestion reactions, in a final concentration of 0.15 μg/mL, were quenched with formic acid (5% final concentration) and centrifuged for debris removal.

### Pre-columns and analytical columns

Reversed phase pre-columns were prepared by first creating a Kasil frit at one end of a deactivated 250 μm ID/360 μm OD capillary (Agilent Technologies, Santa Clara, CA, USA). Kasil frits were prepared by dipping 20 cm capillary in 300 μL Kasil 1624 (PQ Corporation, Malvern, PA, USA) and 100 μL formamide solution, curing at 100°C for 3 h and adjusting the length. Pre-columns were packed in-house (John Yates III's Laboratory, The Scripps Research Institute, La Jolla, CA, USA) with 2 cm of 5 μm ODS-AQ C18 (YMC America, Inc., Allentown, PA, USA) particles from particle slurries in methanol. Analytical reversed phase columns were fabricated by pulling a 100 μm ID/360 μm OD silica capillary (Molex Polymicro Technologies, Austin, TX, USA) to a 5 μm ID tip. The same packing material was packed until 20 cm directly behind the pulled tip. Reversed phase pre-columns and analytical columns were connected using a zero-dead volume union (IDEX Corp., Upchurch Scientific, Oak Harbor, WA, USA).

### LC-MS/MS

Peptide mixtures were analyzed by nanoflow liquid chromatography mass spectrometry using an Easy NanoLC II and a Q Exactive mass spectrometer (Thermo Scientific, Waltham, MA, USA). Peptides eluted from the analytical column were electrosprayed directly into the mass spectrometer. Buffer A and B consisted of 5% acetonitrile/0.1% formic acid and 80% acetonitrile/0.1% formic acid, respectively. The flow rate was set to 400 nL/min. Feeding time saliva samples (1.5 μg per injection) were separated in 155 min chromatographic runs, as follows: 1–10% gradient of buffer B in 10 min, 10–40% of buffer B in 100 min, 40–50% of buffer B in 10 min and 50–90% of buffer B in 10 min. Column was held at 90% of buffer B for 10 min, reduced to 1% of buffer B and re-equilibrated prior to next injection.

The mass spectrometer was operated in a data dependent mode, collecting a full MS scan from 400 to 1,200 m/z at 70,000 resolution and an AGC target of 1 x 10^6^. The 10 most abundant ions per scan were selected for MS/MS at 17,500 resolution and AGC target of 2 x 10^5^ and an underfill ratio of 0.1%. Maximum fill times were 20 and 120 ms for MS and MS/MS scans, respectively, with dynamic exclusion of 15 s. Normalized collision energy was set to 25. The mass spectrometry proteomics data have been deposited to the ProteomeXchange Consortium [54] via the PRIDE partner repository with the dataset identifier PXD003214.

### Data analysis

Tandem mass spectra were extracted from Thermo RAW files using RawExtract 1.9.9.2 [55] and searched with ProLuCID [56] against a non-redundant database containing an Ixodidae database from National Center for Biotechnology Information (NCBI, www.ncbi.nlm.nih.gov) (62,246 entries) concatenated with *Oryctolagus cuniculus* from Uniprot (www.uniprot.org) reference database (21,148 entries) and reverse sequences of all entries. Database sequence redundancies were removed by FastaDBXtractor module from PatternLab for Proteomics platform [57]. Searches were done using Integrated Proteomics Pipeline–IP2 (Integrated Proteomics Applications, Inc., San Diego, CA, USA). The search space included all fully-tryptic and half-tryptic peptide candidates. Carbamidomethylation of cysteine was used as static modification. Data was searched with 50 ppm precursor ion tolerance and 20 ppm fragment ion tolerance.

The validity of the peptide spectrum matches (PSMs) generated by ProLuCID [56] was assessed using Search Engine Processor (SEPro) module from PatternLab for Proteomics platform [57]. Identifications were grouped by charge state and tryptic status, resulting in four distinct subgroups. For each group, ProLuCID XCorr, DeltaCN, DeltaMass, ZScore, number of peaks matched and secondary rank values were used to generate a Bayesian discriminating function. A cutoff score was established to accept a protein false discovery rate (FDR) of 1% based on the number of decoys. This procedure was independently performed on each data subset, resulting in a false-positive rate that was independent of tryptic status or charge state. Additionally, a minimum sequence length of six residues per peptide was required. Results were post processed to only accept PSMs with <10ppm precursor mass error.

### Protein functional annotation and classification

BLASTP searches against several databases were performed to annotate the matched proteins. To check tick proteins identity, the following databases were used: non-redundant (NR), Acari and refseq-invertebrate from NCBI, Acari from Uniprot, the GeneOntology (GO) FASTA subset [58], MEROPS database [59], and the conserved domains database of NCBI [60] containing the COG [61], PFAM[62], and SMART motifs [63]. To check rabbit proteins, the following databases were used: *Oryctolagus cuniculus* and refseq-vertebrates databases from NCBI, *O*. *cuniculus* from Uniprot, the GeneOntology (GO) FASTA subset [58] the conserved domains database of NCBI [60], containing the COG [61], PFAM [62], and SMART motifs [63]. To functionally classify the protein sequences, a program provided by Dr. José M. C Ribeiro written in Visual Basic 6.0 (Microsoft, Redmond, Washington, USA) was used [34]. The functionally annotated catalog for each dataset was manually curated and input in a hyperlinked Excel spreadsheet (S1 and S2 Tables).

### Relative abundance and graphical visualization

To determine the relative abundance of saliva proteins normalized spectral abundance factors (NSAF) were used. The NSAF value was validated as reliable in a label-free relative quantification approach [64–66]. Average NSAF of two or three replicates were used. To determine relative abundance, average NSAF for each protein functional class or an individual annotated protein was expressed as a percent (%) of total NSAF per time point. To visualize relative expression patterns on a heat map, % NSAF values were normalized using Z-score statics using the formula $Z=\frac{X-\mu }{\sigma }$, where *Z i*s the Z-score, *X* is the NSAF for each protein per time point, *μ* is the mean throughout time points, *σ* is the standard deviation throughout time points. Normalized NSAF values were used to generate heat maps using the heatmap2 function from the gplots library in R [67].

### Phylogeny analysis

Amino acid sequences were used to construct a guide phylogeny tree using MacVector 12.7.3 (MacVector Inc Cary, NC, USA) software. Protein sequences were aligned using Muscle method in MacVector under default settings. Subsequently, the tree was constructed using the Neighbor Joining method with uncorrected (“p”) distance setting. To estimate bootstrap values, replications were set to 1000.

## Results and Discussion

### Tick saliva collection

We successfully harvested pilocarpine-induced saliva of *I*. *scapularis* ticks that were partially fed on rabbits for 24, 48, 72, 96 and 120 h as well as those that were apparently engorged but not detached (BD), and those that had engorged and spontaneously detached (SD). During collection of saliva, we observed that saliva of 24 h fed ticks dried up quickly forming flakey white crystal-like residues, and to collect we dissolved these flakes in 2μL sterile phosphate buffered saline (PBS, pH 7.4) batches. On the contrary, saliva droplet of ticks at subsequent feeding stages was visible within seconds to min after pilocarpine injection.

### Protein composition in *I*. *scapularis* tick saliva changes every 24 h

S1 Table lists tick and rabbit proteins that were identified in *I*. *scapularis* saliva. The search of extracted tandem mass spectra against the tick and rabbit protein database using ProLucid [56] and filtering using SEPro [57] produced hits to 769 tick and 130 rabbit proteins respectively with at least one peptide match per protein (S1 Table, please note the different tabs). When subjected to further analysis in BirdsEye View module from PatternLab for Proteomics platform [57], 582 of the 769 tick proteins were determined to be authentic as they were detected in two or all of the three runs, while the remaining 187 proteins detected in only one of the three runs were considered low confidence hits and not further discussed (S1 Table). Of the 130 rabbit proteins that were detected in *I*. *scapularis* tick saliva, 83 met the criteria for authentication. When subjected to auto-annotation [34], 582 tick and 83 rabbit high confidence proteins respectively classified into 24 (Table 1) and 18 (Table 2) functional protein classes. Specifically Tables 1 and 2 summarizes cumulative numbers of proteins that were identified in each functional class, apparent relative abundance at each time point, and time points at where class were not detected [represented by zero (0)].

**Table 1:** Numbers and cumulative relative abundance of tick protein classes in *I*. *scapularis* saliva.

	Feeding Time Point													
	24		48		72		96		120		BD		SD	
Functional Class	Protein	NSAF (%)*	Protein	NSAF (%)*	Protein	NSAF (%)*	Protein	NSAF (%)*	Protein	NSAF (%)*	Protein	NSAF (%)*	Protein	NSAF (%)*
**Anti-oxidant**	1	0.08	6	1.37	3	1.27	3	1.38	3	0.82	6	2.43	32	7.43
**Anti-microbial/ Pathogen recognition domain**	4	17.52	11	16.81	6	10.69	6	5.52	4	5.11	5	6.71	7	6.53
**Cytoskeletal**	6	2.25	16	3.43	9	2.68	10	2.94	1	0.39	6	1.77	27	4.88
**Extracellular matrix/cell adhesion**	2	0.38	5	0.89	3	1.41	1	0.6	0	0	1	0.97	5	1.08
**Glycine-rich protein**	4	1.1	11	2.38	5	3.3	13	4.32	2	1.39	4	5.57	14	4.6
**Heme/iron metabolism**	11	13.61	15	13.46	11	13.12	15	23.94	8	10.97	7	23.74	12	7.74
**Lipocalin**	2	0.3	1	0.06	3	0.95	3	0.55	12	5.82	0	0	7	2.56
**Metabolism, amino acid**	0	0	0	0	0	0	0	0	0	0	1	0.5	20	3.04
**Metabolism, carbohydrate**	2	0.19	4	0.66	1	0.16	4	0.62	4	0.53	5	2.47	20	3.72
**Metabolism, energy**	2	0.62	8	0.97	2	0.24	2	0.24	5	1.32	5	3.23	40	8.13
**Metabolism, intermediate**	0	0	0	0	0	0	0	0	0	0	0	0	1	0.07
**Metabolism, lipid**	1	0.09	2	0.13	1	0.14	1	0.07	0	0	0	0	14	1.12
**Metabolism, nucleotide**	0	0	2	0.1	0	0	1	0.17	1	0.72	3	2.15	14	4.07
**Nuclear regulation**	1	0.6	2	0.76	1	0.55	1	0.76	1	0.42	1	2.48	7	1.33
**Proteinase**	3	0.54	18	3.37	4	0.45	17	5.57	10	2.98	1	0.59	15	1.14
**Proteinase inhibitor**	25	24.72	35	16.86	22	15.02	28	13.13	15	8.11	16	20.82	25	6.72
**Protein export machinery**	0	0	3	0.21	1	0.07	0	0	1	0.62	2	1.3	9	1.42
**Protein modification machinery**	5	2.58	10	3.17	4	5.25	10	2.47	8	1.92	14	8.51	46	13.23
**Protein synthesis machinery**	0	0	1	0.04	1	0.1	0	0	1	0.12	5	2.54	24	3.26
**Proteasome machinery**	1	0.05	2	0.1	1	0.08	1	0.05	1	0.11	1	0.19	10	1.13
**Signal transduction**	1	0.35	3	0.56	0	0	2	0.18	0	0	0	0	8	1.16
**Transcription machinery**	0	0	1	0.1	0	0	1	0.06	0	0	0	0	7	0.6
**Transporters/receptors**	0	0	8	1.07	1	0.03	7	1.25	1	0.02	2	0.15	15	1.57
**TSP of unknown function**	27	35.02	62	33.51	51	44.47	52	36.17	50	58.63	12	13.86	39	13.47
**Total**	98	100	226	100	130	100	178	100	128	100	96	100	418	100

*—Normalized spectral abundance factor

**Table 2:** Numbers and cumulative relative abundance of host (rabbit) protein classes in *I*. *scapularis* saliva.

	Feeding Time Point													
	24		48		72		96		120		BD		SD	
Functional Class	Protein	NSAF (%)*	Protein	NSAF (%)*	Protein	NSAF (%)*	Protein	NSAF (%)*	Protein	NSAF (%)*	Protein	NSAF (%)*	Protein	NSAF (%)*
**Cytoskeletal**	0	0	1	0.65	3	1.2	1	0.05	2	2.04	1	1.15	1	1.28
**Oxidant metabolism/ detoxification**	0	0	0	0	0	0	0	0	0	0	0	0	1	0.16
**Extracellular matrix/cell adhesion**	0	0	1	0.22	1	0.08	2	0.67	0	0	2	1.35	1	0.46
**Fibrinogen**	0	0	0	0	0	0	1	0.25	0	0	1	0.34	3	3.13
**Heme/iron metabolism**	2	74.53	5	39.49	2	2.53	4	17.23	4	41.9	3	15.45	6	16.77
**Hemoglobin/RBC products**	1	7.63	2	7.81	1	1.13	2	9.15	2	16.42	3	50.21	3	39.31
**Immunity**	1	5.62	6	30.04	5	6.71	9	29.59	8	27.59	2	9.38	10	24.59
**Keratin**	5	12.23	5	6.85	6	2.57	7	32.51	5	9.82	5	13.62	1	0.31
**Metabolism, carbohydrate**	0	0	0	0	5	15.54	0	0	0	0	0	0	0	0
**Metabolism, energy**	0	0	0	0	3	6.39	0	0	0	0	0	0	1	0.97
**Metabolism, lipid**	0	0	0	0	1	4.85	0	0	0	0	0	0	0	0
**Metabolism, nucleotide**	0	0	0	0	1	1.21	0	0	0	0	0	0	0	0
**Nuclear regulation**	0	0	2	10.34	2	1.41	1	5.98	0	0	1	2.64	1	4.2
**Proteinase inhibitor**	0	0	1	0.47	0	0	0	0	0	0	0	0	2	0.81
**Protein export machinery**	0	0	2	3.1	4	3.13	1	4.17	2	1.63	2	3.64	2	4.06
**Protein modification machinery**	0	0	1	1.04	10	36.66	1	0.39	1	0.6	1	1.47	3	2.49
**Protein synthesis machinery**	0	0	0	0	4	15.83	0	0	0	0	1	0.74	2	1.43
**Transcription machinery**	0	0	0	0	2	0.74	0	0	0	0	0	0	0	0
**Total**	9	100	26	100	50	100	29	100	24	100	22	100	37	100

*—Normalized spectral abundance factor

**Fig 1:**
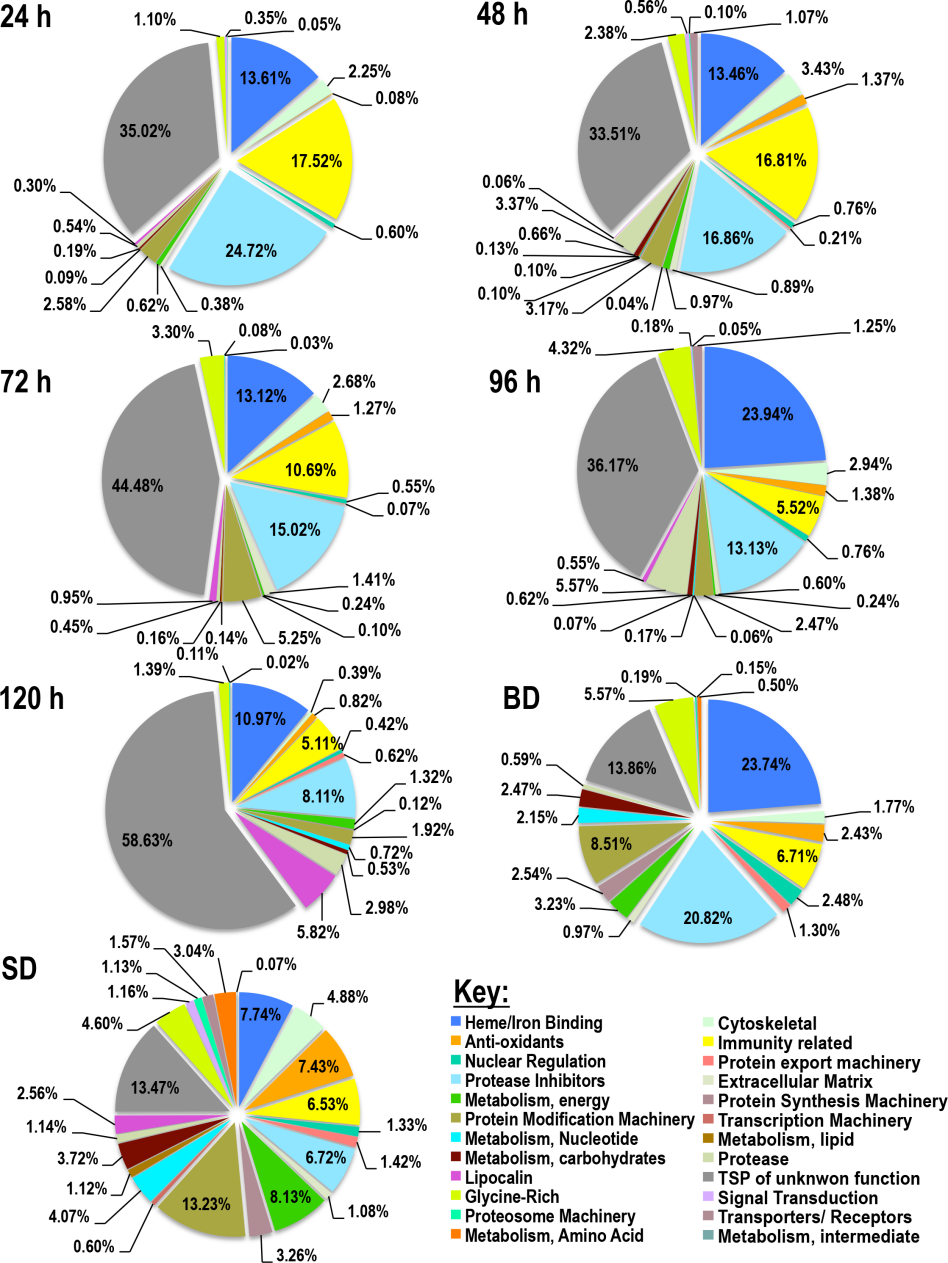
Relative abundance of tick protein classes in *I*. *scapularis* tick saliva during and after feeding. Total normalized spectral abundance factor (NSAF) for each protein class is expressed as a percent of total NSAF per time point. A key is provided listing the 24 classes of proteins identified in tick saliva as tick-derived proteins.

**Fig 2:**
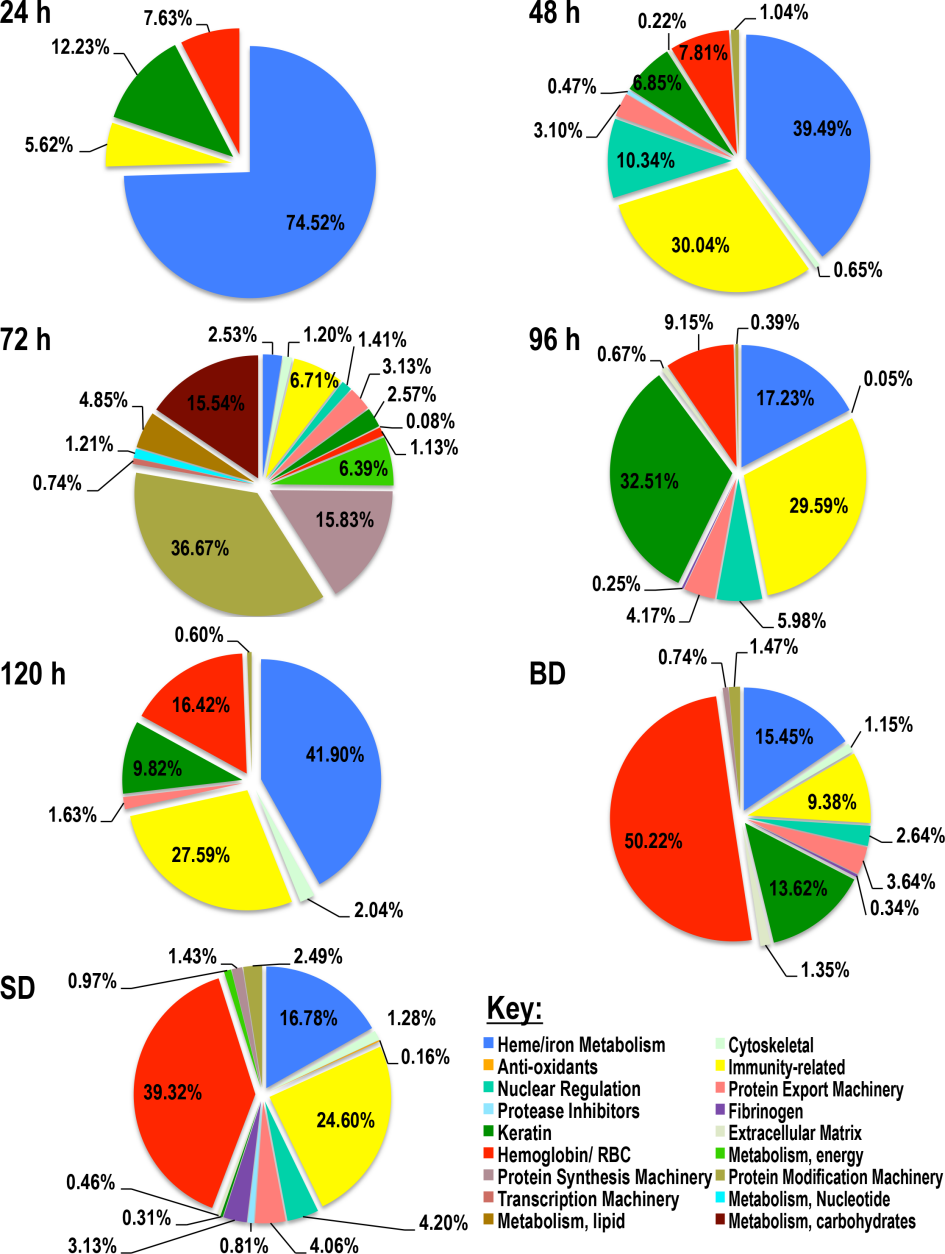
Relative abundance of host (rabbit) protein classes in *I*. *scapularis* tick saliva during and after feeding. Total normalized spectral abundance factor (NSAF) for each protein class is expressed as a percent of total NSAF per time point. A key is provided listing the 18 classes of proteins identified in tick saliva as host-derived proteins.

Figs 1 and 2 gives a snap shot of relative abundance of tick (Fig 1) and rabbit (Fig 2) proteins in *I*. *scapularis* saliva every 24 h. In Fig 1, it is apparent that majority of *I*. *scapularis* tick proteins in this study belong to four predominant functional protein classes starting with proteins of unknown function, followed by protease inhibitors (PI), antimicrobial/immunity related, and heme binding proteins. This is followed by lowly abundant protein classes that account for 1–6% (cytoskeletal, glycine rich, and protein modification machinery) with the remaining protein classes being detected accounted for less than 1%. Of the four major protein classes, relative abundance of proteins of unknown function appear to increase with feeding, accounting for 33–58% of total protein between 24–120 h before dropping to 13% in saliva of fully fed but not detached ticks (BD) as well as fully fed and spontaneously detached. Similarly, heme binding proteins increased from ~14% at 24 h to ~24% at 96 h, before dropping to 10% at 120 h, coming back up to 24% in BD and dropping to 8% in SD. On the other hand, PIs and antimicrobial/immunity related peptides decreased in abundance with feeding with the former dropping from 24.7% at 24 h to 17–8% at 48–120 h respectively, but increasing to 21% in BD and dropping to 7% in SD. Similarly anti-microbial/immunity-related proteins decreased from 18% at 24 h to 17–5% at 48–120 h, before slightly rising to ~7% in BD and SD (Table 1 and Fig 1). Notable protein classes include proteases and lipocalins that appear to increase in abundance with feeding. Protease content increases from 0.5% at 24 h to 3–6% at 48, 96, and 120 h except for 72 h were content was at 0.5%, and 5–6% in BD and SD (Fig 1). Similarly lipocalin content increases from 0.3% at 24 h to ~6% at 120 h, not detected in BD, but accounted for ~3% of protein content in SD. Also notable in Table 1 and Fig 1, tick housekeeping-like proteins appear to increase with feeding.

Fig 2 summarizes relative abundance of rabbit proteins that were detected in *I*. *scapularis* saliva. It is interesting to note that of the 18 protein classes in Table 2, four protein classes: heme/iron, hemoglobin/RBC degradation products, antimicrobial/immunity related, and keratin were found in all time points. It is notable that these four protein classes represented the most abundant rabbit proteins in tick saliva. Except at 72 h where rabbit heme/iron binding proteins accounted for 3%, this protein was among the most predominant in other time points accounting for 17–75% of total rabbit protein content in tick saliva. Similarly, hemoglobin/RBC-related proteins increased from 7.6% at 24 h to 50.2% in BD and 39.3% in SD. Immunity-related proteins of rabbits were most abundant at 48, 96, and 120 h saliva at 30%, 29.6%, and 27.6% respectively (Fig 2). Keratins detected at all time points could signal handling contamination of our samples. Another interesting observation in Fig 2, fibrinogen the precursor to fibrin, which is involved in clot formation was detected toward the end of feeding, 0.25% in 96 h saliva increasing to 3.1% in SD. Could this suggest that the tick ingests fibrinogen during feeding and secretes it back into the host during the detachment phase to promote wound healing? Hard ticks create a wound in host skin from which they suck the blood, however this wound is completely healed when ticks detach.

### Secretion dynamics of selected protein classes during *I*. *scapularis* tick feeding

**Fig 3:**
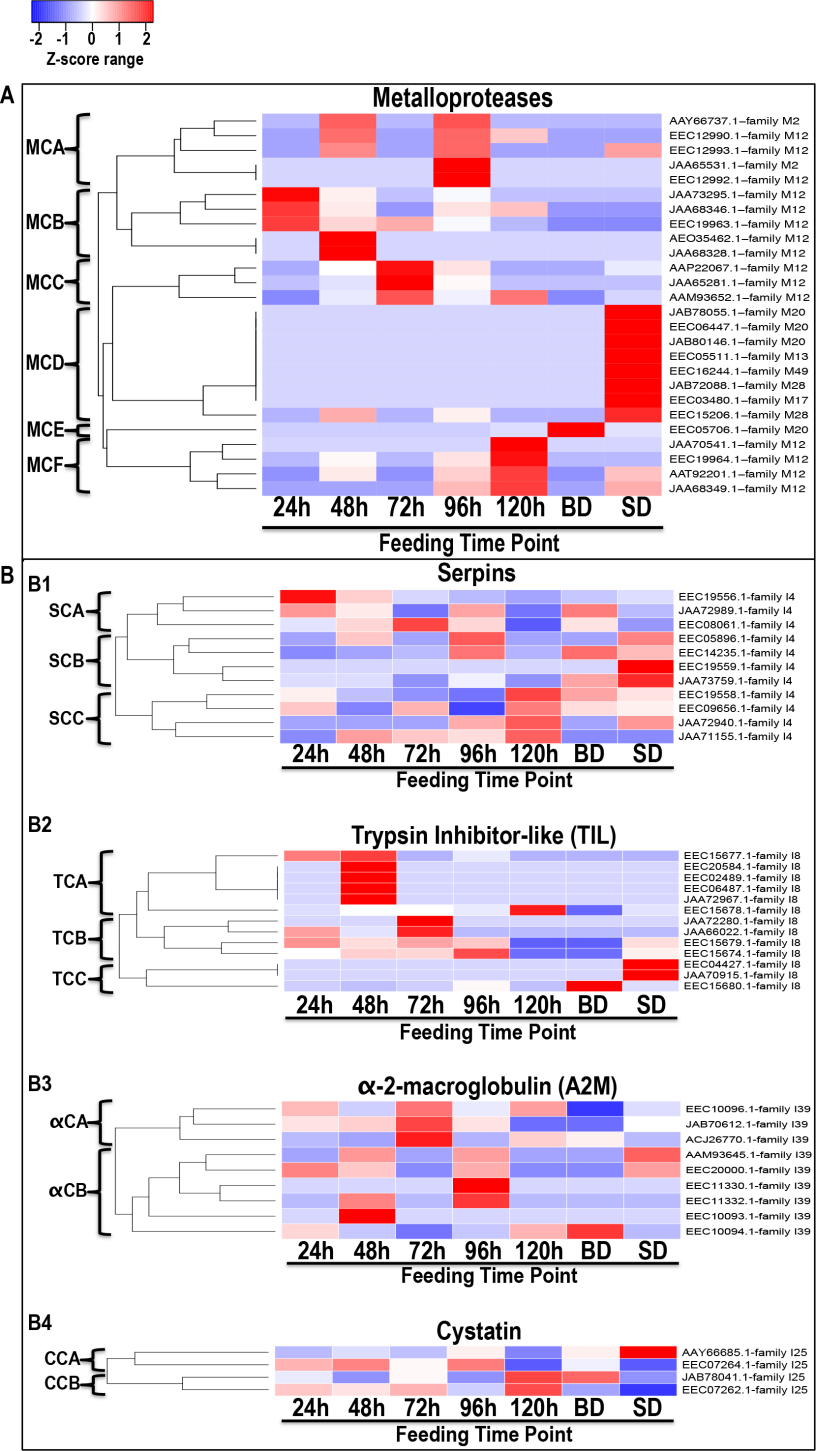
Secretion dynamics of proteases and protease inhibitors in *I*. *scapularis* tick saliva during feeding. Normalized spectral abundance factor (NSAF) for each protein as a proxy for relative abundance is expressed as a percent of total NSAF per time point within each class. Z-scores were calculated and used to generate heat maps as described in materials and methods section. Red color indicates proteins of high abundance and blue color indicates proteins of low abundance, both increasing/decreasing in abundance with color intensity. Dendrograms show protein clustering (C) according to secretion patterns. Branches are labeled starting with the letter of the protein class. Fig 3A (metalloproteases), and Fig 3B (protease inhibitors, B1 = Serpins, B2 = TIL domain protease inhibitors, B3 = α-2-macroglobulin, and B4 = Cystatins) are grouped by functional classes.

**Fig 4:**
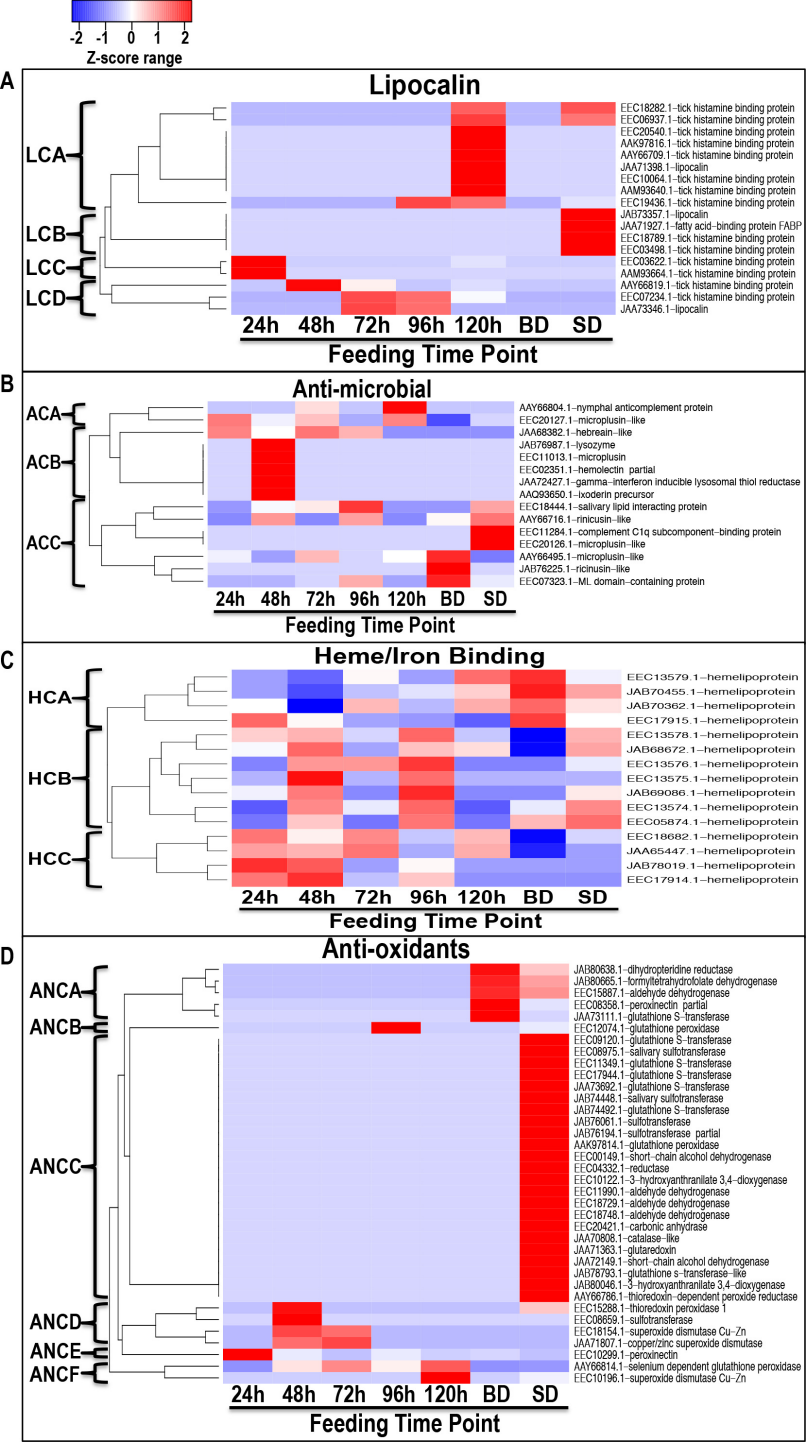
Secretion dynamics of *I*. *scapularis* tick saliva proteins associated with anti-inflammatory (lipocalins), anti-microbial, heme binding, and anti-oxidant functions. Normalized spectral abundance factor (NSAF) for each protein as a proxy for relative abundance is expressed as a percent of total NSAF per time point within each class. Z-scores were calculated and used to generate heat maps as described in materials and methods section. Red color indicates proteins of high abundance and blue color indicates proteins of low abundance, both increasing/decreasing in abundance with color intensity. Dendrograms show protein clustering (C) according to secretion patterns. Branches are labeled starting with the letter of the protein class. Fig 4A (Lipocalins), Fig 4B (Anti-microbial), Fig 4C (Heme binding), and Fig 4D (Anti-oxidants) are grouped by functional classes.

**Fig 5:**
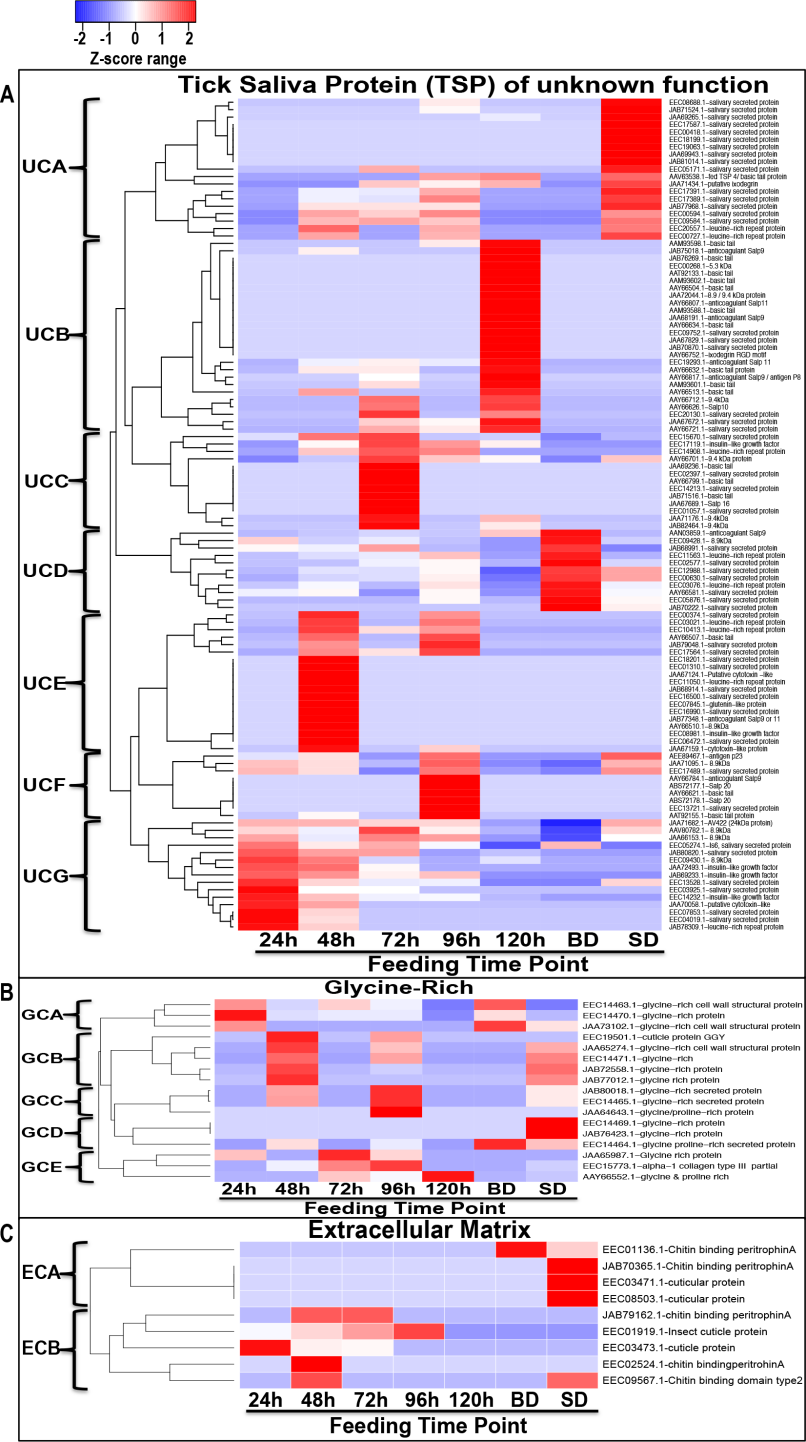
Secretion dynamics of *I*. *scapularis* tick saliva proteins of unknown function(s), glycine rich and proteins associated with the extracellular matrix. Normalized spectral abundance factor (NSAF) for each protein as a proxy for relative abundance is expressed as a percent of total NSAF per time point within each class. Z-scores were calculated and used to generate heat maps as described in materials and methods section. Red color indicates proteins of high abundance and blue color indicates proteins of low abundance, both increasing/decreasing in abundance with color intensity. Dendrograms show protein clustering (C) according to secretion patterns. Branches are labeled starting with the letter of the protein class. Fig 5A (Tick saliva proteins of unknown function), Fig 5B (Glycine-rich), and Fig 5C (Extracellular matrix) are grouped by functional classes.

We are interested in understanding mechanisms that regulate early stage tick feeding, and thus the subsequent discussion of data is biased toward non-housekeeping-like tick derived proteins that were found in saliva from 24/48 h. We have discussed rabbit derived proteins separately, but highlight similarities and differences where appropriate. We used Z-statistics normalization of NSAF values (S3 Table) to develop heat maps in Figs 3–5. These data give insight into relative abundance of specific proteins during feeding: proteases (Fig 3A), protease inhibitors (Fig 3B), lipocalins/tick histamine-binding proteins/fatty acid binding proteins (Fig 4A), anti-microbial/immunity-related (Fig 4B), heme-binding proteins (Fig 4C), anti-oxidants (Fig 4D), proteins of unknown function (Fig 5A), glycine rich proteins (Fig 5B) and extracellular matrix proteins (Fig 5C).

### Majority of proteases in *I*. *scapularis* saliva are metalloproteases

The *I*. *scapularis* genome encodes for at least 233 putatively active and 150 putatively inactive proteases belonging to serine, cysteine, aspartic, metallo, and threonine protease families [68]. In this study we found 33 proteases in four clans: serine- (n = 3), cysteine- (n = 3), aspartic- (n = 1), and metalloproteases (n = 26) (S2 Table). When searched against the Merops database [59], the 26 metalloproteases belong to families M12 (n = 15), M20 (n = 4), M2 (n = 2), M28 (n = 2), M13 (n = 1), M17 (n = 1), and M49 (n = 1) (S2 Table), while serine, cysteine and aspartic proteases are classified in families S1, C1 and A1 respectively. Most of the proteases here are likely associated with tick feeding regulation in that 75% (25/33) were detected between 24–120 h during tick feeding except for seven that were identified only in SD (S2 Table). Nearly 40% of proteases in the *I*. *scapularis* genome are metalloproteases [68]. Whether or not the observation in this study that majority of proteases in *I*. *scapularis* tick saliva are metalloproteases reflects the protease composition in *I*. *scapularis* genome or it is a physiological event, is unknown at this point.

Z-score statistic analysis and visualization of normalized NSAF values of the 26 metalloproteases (Fig 3A) show that M12 and M2 metalloproteases were likely secreted in high abundance between 24–120 h during feeding respectively, which could indicate the importance of these proteins in regulating the first five days to tick feeding. The remaining metalloproteases in families M17, 20, 28 and 49, which were abundant in BD and SD (Fig 3A) are not likely associated with regulating tick feeding events.

The observation that *I*. *scapularis* predominantly secreted family M12 metalloproteases during feeding is suggestive of the importance of this protein class in tick feeding physiology. Emerging evidence indicate that this is the case. A recombinant protein of M12 protease (AAP22067.1, MCC Fig 3A) has gelatinase and fibrin(ogen)olytic activities [69], which is a pro-tick feeding event. In a related study, RNAi silencing of AAM93625.1 (MCC Fig 3A) and AAT92201.1 (MCF Fig 3A) homologs, Metis 1 and 2 (CAO000625 and CAO000626) in *I*. *ricinus* impaired blood meal feeding and egg laying with salivary gland protein extracts of these ticks not affecting host fibrinolysis [70]. In related studies, snake venom M12 proteases were associated with hemorrhaging, edema, hypotension, hypovolemia, inflammation and necrosis [71–73] some of which will promote tick feeding. It will be interesting to characterize the role(s) of tick saliva proteases identified in this study.

### Majority of protease inhibitors in *I*. *scapularis* saliva likely inhibit serine proteases

The first line of host defense to tick feeding such as inflammation, platelet aggregation, blood clotting, complement activation, and cellular immunity are mediated by proteases that are controlled by protease inhibitors (PI). From this perspective, it has been hypothesized that ticks could inject PIs into the host to evade host defense [18, 74–77]. In this study, we identified 43 putative PIs (S1 Table), which according to the Merops database belong in eight families: I2 (Kunitz type serine protease inhibitors, n = 2), I4 (serine protease inhibitors, [serpins], n = 11), I8 (TIL domain serine protease inhibitors, n = 13), I25 (cystatins, cysteine protease inhibitors, n = 4), I31 (thyropins, cysteine protease inhibitors n = 2), I39 (α-2 macroglobulin, A2M, n = 9), I43 (Kazal type serine protease inhibitors, n = 1), and I68 (carboxypeptidase inhibitors, TCI, n = 1) were identified in *I*. *scapularis* saliva (S2 Table). It is notable that 84% (36/43) of PIs were detected in 24 and 48 h saliva (S2 Table), suggesting the potential for these proteins to regulate early stages of tick feeding. The observation here that majority of PIs in this study are likely inhibitors of serine proteases could signal the potential that most host defense pathways to tick feeding are likely serine protease mediated.

Similar to other protein classes in this study, relative abundance of PIs varied every 24 h (Fig 3B). Serpins show three secretion profiles: SCA proteins are abundant in first 48 h and decrease with feeding, SCB are abundant at 96 h and increase in SD saliva, and SCC proteins increase in abundance from 24 to 120 h (Fig 3B1). Similarly, TIL domain PIs segregate in three clusters: those abundant during first 48 h of feeding but decrease with feeding in TCA, increase with feeding between 24–96 h in TCB, and those abundant in BD and SD saliva in TCC (Fig 3B2). In Fig 3B3, alpha-2-macroglobulins segregate in two clusters: those secreted in abundance between 24–120 h in αCA, and 48-SD in αCB. In Fig 3B4, cystatins cluster into CCA for those that increase in abundance with feeding and CCB for those that were secreted in high abundance at the 120 h time point.

There is evidence that some of the PIs identified in this study regulate important tick feeding functions. For instance serpin EEC19556.1 in SCA (Fig 3B1) is 98% identical to AID54718.1, an inhibitor of trypsin and thrombin that also inhibited blood clotting and platelet aggregation [43]. Similarly *I*. *ricinus* serpin ABI94056, the homolog of *I*. *scapularis* serpin EEC14235.1 in this study (Fig 3B1 SCB) is an immunosuppressant, anti-inflammatory, and anti-hemostatic serpin [78–80]. In other studies, *I*. *scapularis* cystatin AAY66685.1 in this study (Fig 3B4 CCA) known as Sialostatin L2 and its close relative Sialostatin L have immuno-modulatory functions, and suppressed cytokine production in absence [81–85] or presence of *B*. *burgdorferi* [86]. It will be exciting to understand role(s) of PIs in *I*. *scapularis* feeding identified in this study.

### Lipocalins/tick histamine-binding proteins (tHBP)/fatty acid binding proteins (FABP)

Lipocalins/HBP and FABPs belong to the calycin superfamily of hydrophobic ligand binding extracellular proteins [87–89]. The lipocalin protein family to which HBPs belong is a large group of proteins that bind and transport small hydrophobic molecules, and also associated with multiple functions including regulation of inflammation through binding of pro-inflammation molecules such as histamine [90–92]. Likewise the FABPs bind and transport hydrophobic ligands including long chain fatty acids, eicosanoids, bile salts and peroxisome proliferators [93]. Tick lipocalins/histamine-binding proteins are thought to be involved with mediating the tick's evasion of the host's inflammation defense through sequestration of pro-inflammatory biogenic amines, lipids, histamine, serotonin and prostanoids [94]. Tick histamine binding proteins (tHBP) are a subset of lipocalins with two histamine-binding pockets [91]. Of the 18 proteins in S2 Table and Fig 4A, 14 are annotated as tHBPs, three as lipocalins, and one as FABP-like. Similar to other proteins, *I*. *scapularis* appears to selectively inject tHBPs/lipocalins into the host at specific time periods, with two tHBPs detected at 24 h in LCC, one at 48 h and three proteins each at 72 h and 96 h in LCD (Fig 4A). The highest numbers of tHBPs/lipocalins were identified at 120 h in LCA (n = 12) of which half were exclusive to this time point (S2 Table and Fig 4A). It is notable that two tHBPs and one each of lipocalin and FABP-like identified in this study were exclusive to SD saliva in LCB, which could suggest that these proteins are involved with events at the end of tick feeding.

A limited number of studies suggest that lipocalins/HBPs/FABP indeed perform tick-feeding functions. Three *R*. *appendiculatus* tHBPs were predicted to suppress inflammation during blood feeding as revealed by its ability to outcompete histamine receptors [90]. In other studies, *D*. *reticulatus* tHBP bound histamine and serotonin [95], and *Ornithodoros*. *moubata* tHBP, referred to as moubatin, demonstrated inhibition of collagen induced platelet aggregation [96]. In a recent study, lipocalins/HBPs/FABPs were identified among 24–48 h *A*. *americanum* immunogenic tick saliva proteins [40] suggesting that these proteins are part of the tick saliva proteins that confer anti-tick resistance in repeatedly infested animals. It is notable that in Radulovic et al., [40], alongside lipocalins/HBPs, a leukotriene B4-like protease was also found among 24–48 h *A*. *americanum* immunogenic tick saliva proteins. It is interesting to note that *I*. *ricinus*, tHBP referred to as LIR6 bound leukotriene B4 [97], a pro-inflammatory mediator and a potent neutrophil chemoattractant.

### *I*. *scapularis* tick saliva anti-microbial proteins

The tick feeding style of tearing up host tissue and sucking up blood from a wounded feeding site exposes the host to microbial infections. From this perspective ticks were postulated to inject anti-microbial peptides into the feeding site to prevent the feeding site from being infected [23, 48]. Multiple anti-microbial peptides have been characterized in ticks, a majority of which are defensins [98–106], microplusin/microplusin-like [107–109] and hebreain/hebreain-like [110]. In this study seven of the 15 anti-microbial peptides in S2 Table and Fig 4B are microplusin-like, a single lysozyme, and the rest, are characterized by pathogen-recognition domains (n = 7). Fig 4B shows three secretion patterns, where ACA proteins were abundant during 24–120 h, ACB were only present in 48 h and ACC proteins increase from 48–96 h but highly abundant in BD and SD saliva. Except for microplusin [107], which was shown to stop *Micrococcus luteu*s and *Cryptococcus neoformans* growth [111], nothing is known on the role(s) of most of the anti-microbial peptides in this study. It is notable that majority of anti-microbial peptides in this study are apparently injected into the host within the first 48 h of feeding (n = 11) (S2 Table and Fig 4B). Understanding functions of some of these antimicrobial peptides will reveal microbes that *I*. *scapularis* want to keep out of the feeding site.

### Heme-binding proteins

When fully fed, hard ticks are estimated to imbibe host blood that is more than 100 times the their original weight [112]. Catabolism of this huge amount of blood generates high amounts of iron and heme [113–115]. Both iron and heme are needed for normal cell function [113, 114]. However, if left unsecured, both iron and heme can cause cell damage through promotion of oxidative stress [116, 117]. Ticks are postulated to prevent iron and heme mediated tick cell damage through expression of iron and heme binding proteins, which play two roles: bind and distribute to cells for normal physiology, and sequester excess iron or heme and prevent oxidative stress triggered cell damage [115].

One of the most notable observations in this study is that although heme-binding proteins represented ~2.6% (15/582) of proteins identified, they accounted for ~11–24% of total protein abundance (Table 1 and Fig 1). This could suggest that heme metabolism is potentially a “must-not-fail” tick physiological function. The observation that all 15 heme binding proteins in this study are likely injected into the host from within 24–48 h of the tick starting to feed (S2 Table) suggests that this mechanism is important from the start of tick feeding. In Fig 4B three secretion patterns are observable: HCA increases in abundance in 120 h-BD proteins, HCB abundant in 48 and 96 h, and HCC abundant in first 48 h but decrease with feeding. It is notable that the five heme binding proteins that were detected at all time points (S2 Table) cluster together in HCA (Fig 4C) with the exception of EEC13578.1. These proteins account for up to 38% of total NSAF within this class, which could suggest their significance in tick feeding physiology.

It is interesting to note that both iron and heme-binding proteins were also detected in high abundance in saliva of *D*. *andersoni* [51], *R*. *microplus* [50], and *H*. *longicornis* [52]. However only the latter was detected in this study. Whether or not this is unique to *I*. *scapularis* or that iron-binding proteins were injected at below detectable levels needs further investigation. Published evidence has suggested that the tick may detoxify heme/iron through sequestration in digestive cells (hemosomes) [118, 119] and hemolymph [120–122]. Data in this study and others [40, 41, 50, 52, 123] that show secretion of heme binding proteins in tick saliva suggest a third possibility of eliminating heme through tick saliva. Given that heme has pro-inflammatory functions[124], secretion of these proteins in tick saliva may be associated with heme sequestration, and thus allowing tick evasion of the host's inflammation defense. Iron sequestration is among the mammalian host's anti-microbial defense. To counter the host's iron sequestration defense, microbes have developed elaborate ways to bind iron from the environment [125–127] and directly uptake heme, which is then digested to release associated iron [128]. From this perspective it is possible that secretion of heme binding proteins is the tick's strategy to make heme available to transmitted pathogens at the tick-feeding site. It is important to note here that *B*. *burgdorferi*, the most important *I*. *scapularis* transmitted human TBD agent, may not require iron to colonize the host [129].

### Anti-oxidants

Tissue injury caused by tick feeding such as disrupting host tissue and then sucking blood from the wounded area will lead to production of reactive oxygen species (ROS), which will in turn damage host tissue and/or transmitted TBD agents [130, 131]. Thus, it is expected that ticks would inject anti-oxidants into the feeding site as observed in this study. Fig 4D summarizes relative abundance of 36 putative anti-oxidant proteins, 23 of which were identified only in SD saliva (S2 Table), and are likely associated with events toward end of tick feeding. The remaining 13 proteins were identified between 24 h-BD and are likely associated with tick feeding regulation. The heat map in Fig 4D show that different anti-oxidants were detected in high abundance at different time points: ANCA in BD and SD, ANCB at 96 h, ANCC in SD, ANCD at 48 and 72 h, ANCE at 24 h and, ANCF at 120 h. It is interesting to note that some of the data in this study are consistent with previous observations. Glutathione peroxidase (AAK97814.1) previously found among immuno-dominant proteins in engorged *I*. *scapularis* [132] is among the 23 anti-oxidants that were found in SD saliva only (S3 Table and Fig 4D).

The role(s) of antioxidants in tick physiology remain mostly unknown. In a recent study, thioredoxin peroxidase gene expression increased in organs of *B*. *burgdorferi* infected *I*. *ricinus* ticks [133] suggesting involvement in tick and pathogen interaction. It is interesting to note in this study thioredoxin peroxidase protein in non-infected ticks decreased with feeding (S3 Table). It will be interesting to determine if anti-oxidant proteins identified from this study may play roles at the tick-host interface in TBD acquisition and transmission.

### *I*. *scapularis* tick saliva proteins of unknown function

More than 30% of tick sequences in public databases are of unknown function [30, 32–37, 48, 134–138]. In this study we have identified 129 tick saliva proteins (TSP) of unknown function (S2 Table). For clarity secretion profiles of the 112 TSPs of unknown function are summarized in Fig 5A, while the remaining 17 glycine-rich proteins, which are thought to be involved in tick cement formation [139] are shown in Fig 5B. It is interesting to note that in S2 Table, 93.7% (105/112) of TSPs were detected in 24–120 h saliva which could indicate that these proteins are important to tick feeding physiology. The remaining 6.3% (7/112) were exclusive to BD and SD stages and are likely associated with events towards end of feeding. Some proteins were found at one time point: 48 (n = 12), 72 (n = 7), 96 (n = 5) and 120 h (n = 14) saliva (S2 Table). More than half (n = 62) of TSPs of unknown function were detected within the first 48 h of feeding. These could be crucial for tick feeding initiation and progression. Patterns in Fig 5A suggest that the tick may potentially selectively inject different proteins into its host every 24 h. In this way, the tick could successfully evade host immunity and acquire a blood meal. Seven clusters (UCA-UCG) of TSP of unknown function are observed (Fig 5A). Most notable is that TSP of unknown function that are highly abundant at 24 h (UCG Fig 5A), decrease with feeding indicating that these proteins could serve as pivotal proteins in commencing the tick feeding process. Other secretion patterns include proteins that are abundant at 48, 72, 96, and 120 h in UCE, UCC, UCF, and UCA respectively, as these proteins could be important in maintaining different phases of the tick feeding process. Proteins in UCD and UCA could play important roles towards end of feeding such as in wound healing and detachment from its host or serve as markers for completion of tick feeding.

Like several other hard ticks, *I*. *scapularis* ticks secrete cement to securely anchor onto host skin during the prolonged tick-feeding period [1, 139, 140]. Chemical analysis studies have shown that tick cement has a high content of glycine-rich proteins [139]. On this basis, we speculate that glycine rich proteins in S2 Table could be associated with tick cement formation. The first layer of the tick cement cone is deposited within 5–30 min of the tick attaching, while the second layer starts to form from 24 h post attachment [139]. It is interesting to note that majority (n = 13) of the glycine rich proteins were identified in high abundance in 24 and 48 h saliva (S3 Table and Fig 5B). Secretion patterns of glycine rich proteins shown in Fig 5B suggest that the tick alternates secretion of these proteins during feeding. Most notably the proteins in GCA are most abundant in 24 h, GCB in 48 h, GCC in 96 h, GCD in BD-SD, and GCE in 72–120 h saliva (Fig 5B). The importance of glycine rich proteins detected in abundance towards the end of feeding is unknown at this point. However, there is a possibility for these proteins representing products of degenerated salivary glands. It will be interesting to determine the function of these proteins towards the end of feeding.

**Fig 6:**
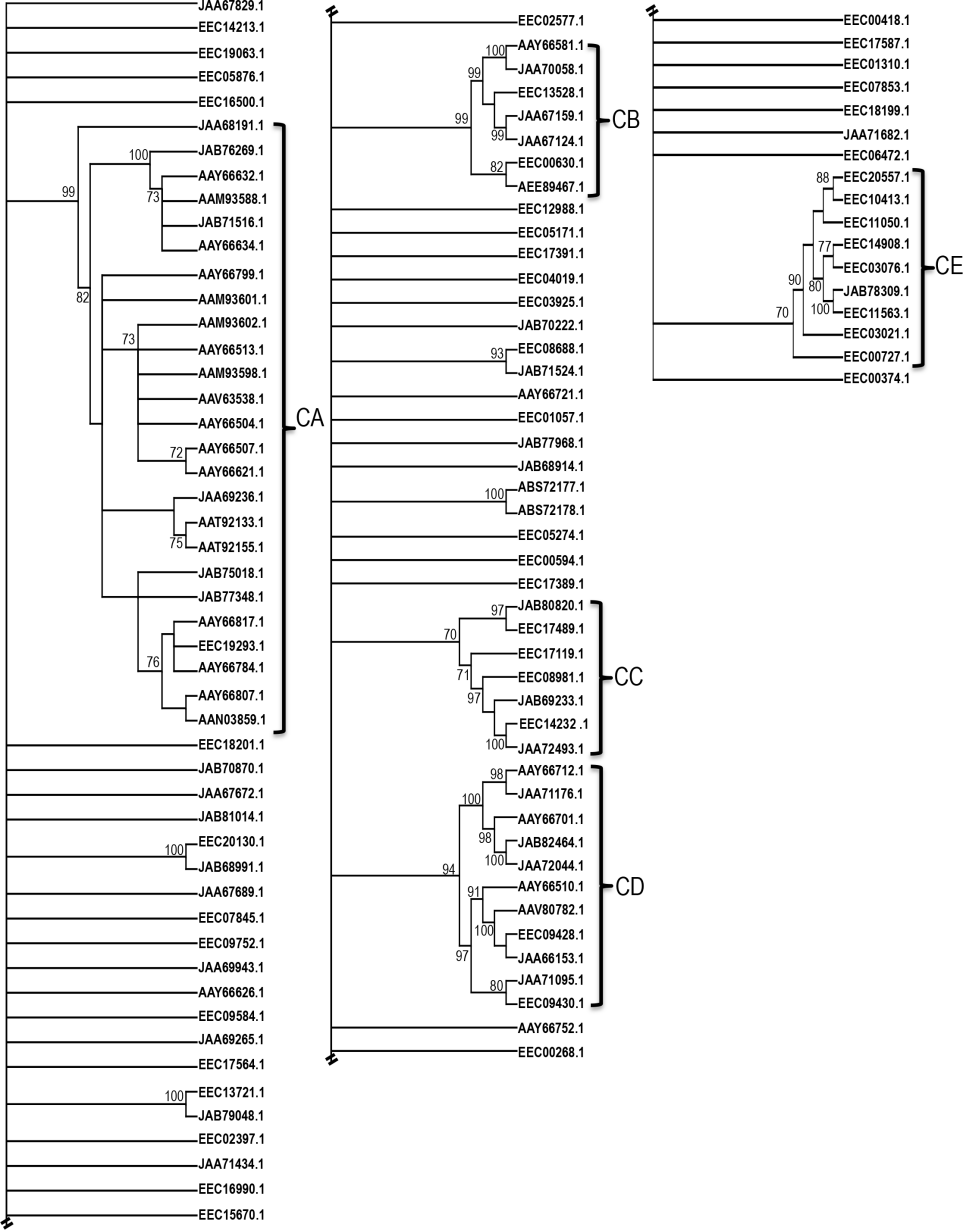
Phylogeny analysis of tick saliva protein of unknown function identified in *I*. *scapularis* saliva during and after feeding. A guide phylogeny tree of tick saliva proteins (TSPs) of unknown function sequences was constructed using the Neighbor Joining method with bootstrap replicates set to 1000. Number at each node represents bootstrap values that signify the level of confidence in the branch. Five main groups cluster as: (CA) basic tail or tailless proteins, (CB) GPIIb/IIIa antagonist, (CC) insulin binding-like proteins (CD) 7-9kDa proteins and (CE) leucine rich proteins.

When subjected to phylogeny analysis, 40.2% (45/112) of TSP of unknown function are unique in that they segregate individually, followed by 7.1% (8/112) that cluster in pairs, and the remaining 52.7% (59/112) segregate in five clusters (C) A-E (Fig 6). According to previously described classifications of *I*. *scapularis* proteins [136], CA, CB, and CD clusters are respectively classified as basic tail (group 1, n = 15) or tailless proteins (group 2, n = 10), GPIIb/IIIa antagonist (group 9, n = 7), and 7–9 kDa family (group 7, n = 11). TSPs in CC cluster (n = 7) have insulin binding-like proteins motifs [141], while CE cluster proteins are leucine rich (n = 9) as revealed by sequence inspection. On the basis of amino acid motifs, Ribeiro et. al., [136] classified basic tail and basic tailless proteins into types I-III. Of the 25 CA proteins, 44% (11/25) and 20% (5/25) fit to basic tail types I and II protein respectively, and the remaining 36% (9/25) fit to basic tailless proteins.

**Fig 7:**
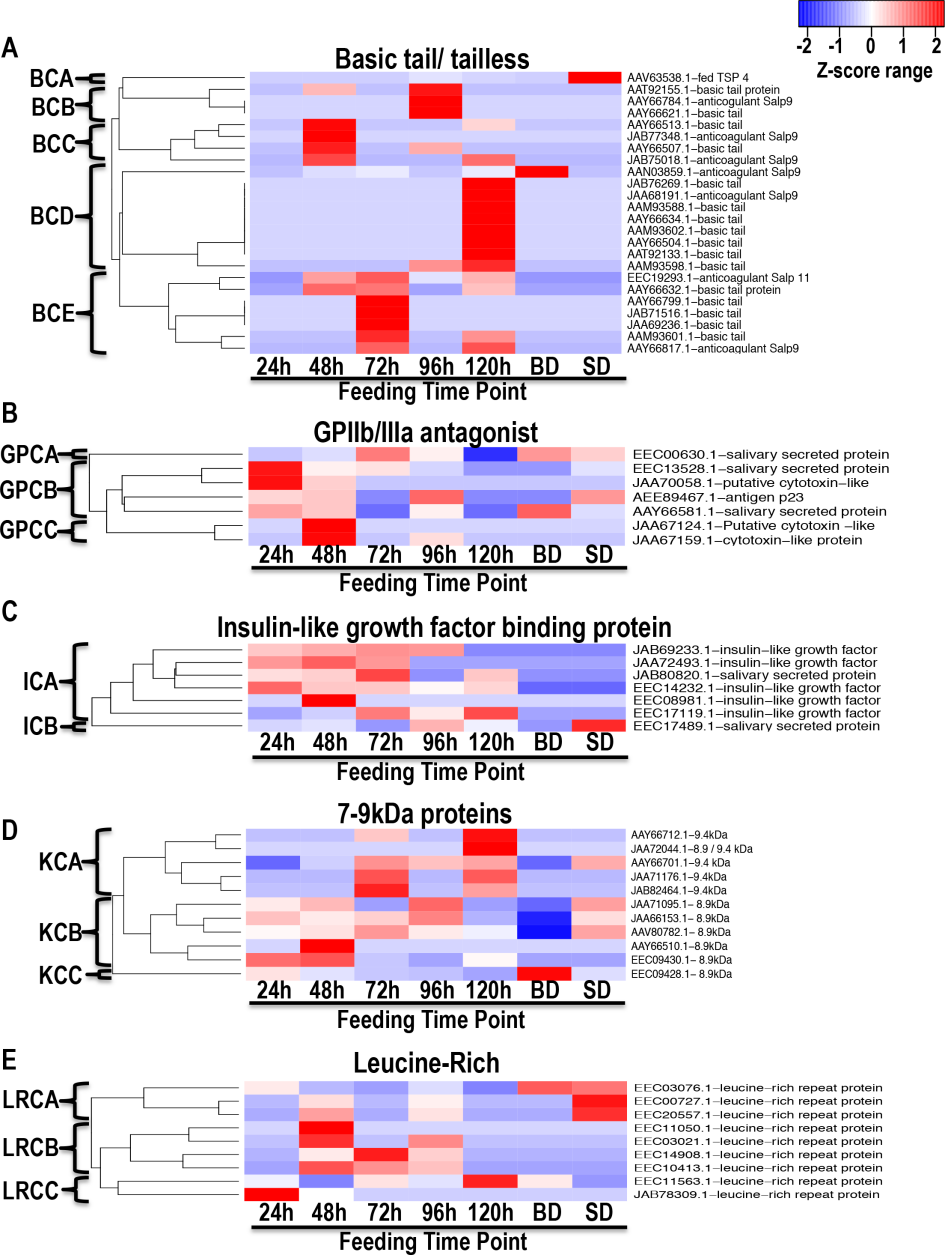
Relative abundance of tick saliva proteins (TSP) of unknown function during and after feeding. Normalized spectral abundance factor (NSAF) for TSP that segregated together in Fig 6 was subjected to Z-score statistic analysis and used to generate heat maps as described in materials and methods section “Relative abundance and graphical visualization”. Red color indicates proteins of high abundance and blue color indicates proteins of low abundance, both increasing/decreasing in abundance with color intensity. Clustering patterns of dendrograms were based on similarity in secretion patterns. A = basic tail or tailless proteins, B = GPIIb/IIIa antagonist, C = insulin-like growth factor binding proteins, D = 7-9kDa proteins, and E = Leucine-rich proteins.

An interesting observation from our data is that proteins that segregated together in the (Fig 6), were identified at different time points (Fig 7A–7E) suggesting that the tick could be selectively secreting these proteins during feeding. Similar to Figs 3–5, we used Z-statistics normalization of NSAF values (S3 Table) to develop heat maps in Fig 7A–7E. Basic tail or tailless proteins segregated into five clusters according to secretion patterns starting with the lone protein in BCA that is abundant in SD, followed by proteins in BCB, BCC, BCD and BCE that are respectively abundant in 96, 48, 120, and 72 h saliva (Fig 7A). Likewise in Fig 7B, GPIIb/IIIa antagonist protein cluster in three groups: abundant from 72 h (GPCA), 24 and 48 h (GPCB), and 48 h only (GPCC). In Fig 7C, except for one protein, which is abundant in SD saliva (ICB), majority of these proteins are abundant in 24–72 h saliva (ICA). In Fig 7D and 7E, 7–9 kDa and Leucine rich proteins were identified at variable levels throughout feeding.

Putative GPIIb/IIIa in GPCB (Fig 7B) cluster are characterized by "RGD" motif and can potentially block platelet aggregation by blocking activated platelets from binding to fibrinogen [142]. In a recent study peptides containing the "NGR" motif prevented resting platelets to bind to fibrinogen [143]. It is interesting to note that four (AAY66799, AAY66507, AAY66621, and AAY66504) basic tail saliva proteins have this motif. Whether or not these proteins can functionally block platelet aggregation of resting platelets needs verification. If functional, these could play key roles in tick feeding success in that at the start of tick feeding, the tick will encounter resting platelets. Surprisingly none of the four NGR motif proteins were detected in 24 h saliva when we expect resting platelets at the feeding site. Interestingly, except for AAY66507.1 (UCE) detected in 48 and 96 h saliva, the other three were detected at single time points: AAY66799.1 at 72 h (UCC), AAY66621.1 at 96 h (UCF), and AAY66504.1 at 120 h (UCB).

### *I*. *scapularis* tick saliva extracellular matrix-like proteins

Similar to glycine-rich proteins, extracellular matrix proteins likely participate in tick cement formation and/or cell adhesion function. In this study we found 9 extracellular proteins (S2 Table) that included cuticle and chitinase-like proteins. Two secretion patterns are observed in Fig 5C, where ECB proteins were abundant from 24–96 h and ECA proteins were abundant in BD and SD saliva. No proteins were detected in 120 h saliva from this class. It is interesting to note that both the active and inactive forms of chitinase were identified in the first 48 h. The former is highlighted by a peritrophin-A chitin-binding domain, which is involved in remodeling the chitinous tick exoskeleton, particularly the mouthpart [144, 145]. The latter is highly identical to *A*. *americanum* tick feeding stimuli responsive acidic chitinase [134], which when silenced by RNAi caused ticks to loosely attach onto host skin [146]. Blast2seq alignments revealed that the two *I*. *scapularis* inactive chitinases (EEC01936.1 and JAB70416.1) identified in both 24 and 48 h saliva are respectively 64 and 65% identical to *A*. *americanum* inactive chitinase (AIR95100.1). Whether or not *I*. *scapularis* inactive chitinases serves similar function during tick feeding needs further investigation.

### Housekeeping proteins and other TSPs

In addition to anti-oxidants discussed above, housekeeping-like proteins identified in this study include those associated with metabolism of lipids (n = 15), carbohydrates (n = 20), intermediate (n = 1), energy (n = 45), nucleotides (n = 14) and amino acids (n = 20) (S2 Table). Others are classified as cytoskeletal (n = 32), proteasome machinery (n = 10), protein modification (n = 49), protein synthesis (n = 24), protein export (n = 10), nuclear regulation (n = 7), signal transduction and apoptosis (n = 8), transcription machinery, (n = 8), and transporters and receptors (n = 19) (S2 Table). Cumulative NSAF as an index for protein abundance suggests that majority of housekeeping-like proteins were secreted toward the end of tick feeding in BD and SD saliva, respectively (Table 1).

The tick salivary gland starts to degenerate toward the end of tick feeding and is almost completed within four days of the tick detaching [140, 147]. Given that most housekeeping genes function inside the cell, one may argue that the high abundance of these proteins in BD and SD saliva may represent progressive SG degradation toward end of tick feeding. However, recent immuno-screening of phage display expression libraries with antibodies to 24 h *I*. *scapularis* [41] and 24–48 h *A*. *americanum* [40] tick saliva proteins that identified housekeeping-like indicates that secretion of some of the housekeeping proteins starts way before tick salivary gland degeneration, and thus, these proteins likely play important role(s) in tick feeding regulation.

One remarkable tick adaptation is that although ticks feed from a wounded area in the host's skin, the feeding site is completely healed when ticks complete feeding and detach from host skin. There is a possibility that some of the proteins identified in BD and SD could be associated with speeding up wound healing. It is interesting to note that some cytoskeletal proteins including actin [148–152], profilin [153, 154], alpha tubulin [154], calponin [155, 156], non-muscle myosin [149, 157, 158], thymosin [159], and tropomyosin [160] identified at high abundance in BD and SD saliva were associated with different aspects of wound healing. Could secretion of these proteins at high abundance be the tick's way to help the host heal?

### Host proteins in *I*. *scapularis* saliva

When ticks feed on blood, they uptake thousands of host proteins. The observation in this study that *I*. *scapularis* secreted 83 out of thousands of host proteins suggests that the tick has a mechanism to selectively secrete host proteins in its saliva. Similar to secretion dynamics of tick-derived proteins, the tick appears to selectively secrete different rabbit proteins at different tick feeding time points (S1 Table). It is potentially possible that similar to tick-derived proteins, host proteins in tick saliva perform functions that are unique to different tick feeding phases. Proteins identified from 24/48 h saliva and other time points (immunity/antimicrobial function, heme/iron metabolism, hemoglobin, nuclear regulation, extracellular matrix, and collagen alpha-1 chain), likely aide the tick to feed. On the other hand, proteins identified in BD and SD saliva such as fibrinogen and protease inhibitors are likely associated with events toward the end of tick feeding. For instance, functionally annotated antimicrobial peptides: antimicrobial protein CAP18 [161, 162] identified in all samples except 120 h saliva, neutrophil gelatinase-associated lipocalin [163, 164], neutrophil granule protein [165–167], protein S100-A12 [168], neutrophil antibiotic [165], and lysozyme C [169, 170] that were identified in 48 h and other stage saliva (S2 Table) could aid the tick to clear microbes from the tick feeding site. It is interesting to note that we identified both tick- and rabbit- derived antimicrobial peptides at the same time points. It is most likely that these antimicrobial peptides target different microbes with tick-derived proteins clearing tick-derived microbes, whereas host-derived proteins clear microbes from the host. Cell free hemoglobin (Hb) was shown to possess antimicrobial activity through oxidative shock [171, 172], and thus there is a possibility that Hb detected in tick saliva could be providing antimicrobial function [173, 174] at the tick-feeding site. In another study peptides derived from hemoglobin digestion by tick proteases have been described as antimicrobial peptides [173, 175, 176]. It is also possible that secretion of Hb could just be an indication of blood meal digestion.

Similar to ticks (Fig 1), rabbit derived heme/iron metabolism associated proteins were the highly abundant at all time points (Fig 2). A notable difference is that whereas we exclusively identified heme-binding proteins for ticks, we identified a majority of iron binding proteins for rabbits (n = 6) and one heme binding protein (S2 Table). Could this mean that, *I*. *scapularis* uses host proteins to remove excess iron though its saliva? If so, it could be that *I*. *scapularis* tick-derived heme binding proteins are responsible for removing heme, but the tick engages host-iron binding proteins to remove excess iron. Except for haptoglobin [177], which was detected in SD saliva, all other iron binding proteins: serum albumin, histidine rich glycoprotein, lactotransferrin, and serotransferrin as well as the heme binding protein, hemopexin and serum albumin were identified from 24/48 h saliva and other stages during feeding (S2 Table). Based on our data, *I*. *scapularis* apparently could use host proteins to eliminate excess iron from the host starting within 24–48 h. It is also interesting to note that human serum albumin was shown to suppress tumor necrosis factor-alpha (TNF) and complement component C5a triggered neutrophil respiratory burst [178, 179]. It is possible the increased concentration of serum albumin at the tick-feeding site could serve other functions. Given that the host uses iron sequestration as the defense mechanism against microbes [180–183], it is possible that the tick's manipulation of the host to pump back iron into the feeding site could be an adaptation to aide TBD agents to colonize the host, with exception of organisms such as *B*. *burgdorferi*, which do not need iron for proliferation [129].

It is interesting to note that in this study we detected rabbit fibrinogen in BD and SD saliva. Fibrinogen is the source for fibrin needed to strengthen the blood clot [184, 185]. Could it be that the tick pumps back fibrinogen into the host to aide in sealing off the feeding site at the end of tick feeding? Given that high abundance of keratins are expressed in the skin [186, 187], there is a possibility that keratin proteins identified in *I*. *scapularis* tick saliva could be due to sample handling or rabbit skin contamination. It is important to note that all keratin types that were identified in *I*. *scapularis* tick saliva in this study are associated with different layers of the skin [188, 189], and thus there is a high chance we identified remnants on tick mouthparts. However the eight keratin proteins identified in this study represent less than a quarter of the 27 skin keratins [189]. Does the tick selectively inject keratins, and for what purpose is an interesting question for future research.

### Conclusions and future perspectives

The unique contribution of this study is that, we have for the first time attempted to identify tick- and host- derived proteins that are found in *I*. *scapularis* tick saliva every 24 h through the first five days of feeding as well as toward the end of feeding. This study provides identities of *I*. *scapularis* tick saliva proteins associated with regulation of: (i) early tick feeding events such as tick attachment onto host skin and creating the feeding lesion, which precede tick transmission of TBD agents, (ii) slow feeding phase when most TBD agents are transmitted and the tick prepares for rapid feeding phase, and (iii) rapid feeding phase when the tick feeds to repletion and detaches from the host. The impact of these data on future in depth tick feeding physiology studies is vast. For instance, transmission of most TBD agents occur at least 36–48 h post tick attachment [190–194]. What happens if we immunize against 24–48 h tick saliva proteins, is TBD agent transmission stopped? On the other hand we have identified proteins that were apparently secreted at all time points. In future studies, it would be interesting to determine if these proteins regulate "must have" pathways? It will be interesting to validate the importance of such proteins using the RNAi silencing approach. Some proteins were found at single, two or three time points, could these regulate functions unique to that tick-feeding period?

An interesting recurring pattern observed in these data is that some functionally similar but antigenically unique proteins were identified at different feeding time points. We speculate that this could be the tick's strategy to protect essential pathways from immune response attack. For instance, host immune response against 24 h proteins will not affect functions of functionally similar but antigenically unique proteins at later feeding time points. Essentially the host immune defense against tick feeding will restart every so often, and in the end it will not be effective. In this way key tick feeding physiological functions will continue uninterrupted. Could this mechanism be the tick's equivalent to antigenic variation used by parasites such as Trypanosomes to evade host immunity [195–197]? What happens if we target as a cluster of functionally similar but antigenically unique proteins that are injected into the host at different time points?

We would like to caution the reader on the inherent limitations of this study. First, in LC-MS/MS approaches, there is a possibility that predominant proteins will mask discovery of lowly expressed but important proteins, and thus the list of *I*. *scapularis* tick saliva proteins presented here may not be exhaustive. Second, we sequenced proteins in saliva that was collected by pilocarpine stimulation, and whether or not all detected proteins are secreted under physiological conditions remains to be investigated. However, we are encouraged by our findings that 13% (76/582) of *I*. *scapularis* tick saliva proteins in this study were reported in other tick saliva proteomes and immuno-transcriptome studies (S4 Table). Of the 76 proteins, 12 and 13 proteins were found among tick saliva immunogenic proteins that bound antibodies to 24 h *I*. *scapularis* [41] and 24–48 h *A*. *americanum* tick saliva proteins [40] respectively. Additionally one protein was identified in *I*. *scapularis* nymphs as an immunogenic protein that bound to human serum from exposure to tick bites [198]. The remaining proteins were found in saliva proteomes of *R*. *microplus* (n = 28, [50]), *H*. *longicornis* (n = 22, [52]), *D*. *andersoni* (n = 2, [51]), *O*. *moubata* (n = 5, [53], sequencing of *I*. *scapularis* tick saliva by Edman degradation (n = 4) [48], and others were verified as secreted in western blotting studies [42, 43, 199–204].

*I*. *scapularis* proteins in S4 Table could represent highly conserved tick saliva proteins that regulate important functions, which if disrupted could affect the tick. These proteins could represent priority candidates in future studies. We would like to note that some of the protein sequences in this study are from *I*. *ricinus* and other tick species. Majority of these protein sequences have homologs in *I*. *scapularis*, which were eliminated as redundancies when we collapsed the local database. *I*. *ricinus* proteins in this study represent highly conserved proteins among *Ixodes* spp ticks.

## Supporting Information

S1 Table Protein count, spectral count, and NSAF raw data. Two tabs contain the raw data for tick and rabbit derived proteins.
(XLS)

S2 Table Normalized percentage of NSAF values. Two tabs contain the normalized percentage of NSAF for tick and rabbit derived proteins.
(XLS)

S3 Table Standardized NSAF values by Z-scores. Z- score of each functional class is represented in separate tabs.
(XLS)

S4 Table *Ixodes scapularis* tick saliva proteins found in immuno-transcriptomes and other tick saliva proteomes. Proteins in this study were compared to previously published studies and found that 76 tick saliva proteins in this study are also secreted by other tick species.
(XLSX)
